# The Inhibition of Serine/Threonine Protein Phosphatase Type 5 Mediates Cantharidin Toxicity to Control *Periplaneta americana* (L.)

**DOI:** 10.3390/insects11100682

**Published:** 2020-10-08

**Authors:** Hong Sun, Yifan Li, Xinyu Li, Yalin Zhang

**Affiliations:** Key Laboratory of Plant Protection Resources & Pest Management of the Ministry of Education, College of Plant Protection, Northwest A&F University, Yangling 712100, China; sunhong@nwafu.edu.cn (H.S.); liyifanshr@nwafu.edu.cn (Y.L.); lixinyu8023@nwafu.edu.cn (X.L.)

**Keywords:** *Periplaneta americana*, Cantharidin, serine/threonine protein phosphatase

## Abstract

**Simple Summary:**

The American cockroach, *Periplaneta americana* (L.), is a worldwide common sanitary pest. Controlling cockroaches mainly relies on chemical insecticides. However, the irrational and extensive use of insecticides has resulted in increasing resistance in cockroaches. Therefore, alternative agents are urgently needed. Cantharidin (CTD) is an insect defensive toxin that has a significant toxicity against a broad range of pests. In this study, we evaluated the bioactivity of CTD and its derivative Norcantharidin (NCTD) against *P. americana* to determine their potential for controlling *P. americana*. CTD showed a significant ingestion toxicity against *P. americana* (LC_50_ = 50.9 μg/mL). To further explore the reason for the toxicity of CTD against *P. americana*, we cloned and expressed the serine/threonine protein phosphatase type 5 in *P. americana* (PaPP5) and performed inhibition assays of CTD and NCTD on serine/threonine protein phosphatases (PSPs) and PaPP5. The inhibition assays demonstrated that both CTD and NCTD had inhibitory effects on PSPs and PaPP5. The inhibitory capacity of CTD was superior to that of NCTD in both the PSPs and PP5 inhibition assays. These findings contribute to our understanding of CTD as a biorational pesticide to control *P. americana* and provide new insights into insecticide development using PP5 as a target.

**Abstract:**

The American cockroach, *Periplaneta americana* (L.), is a notorious urban pest. It has developed insecticidal resistance to commonly used insecticides. Cantharidin (CTD) is a defensive toxin derived from blister beetles. It has been verified to have insecticidal toxicity in a range of pests. In this study, we determined the ingestion toxicity of CTD and norcantharidin (NCTD) to *P. americana* to test whether they had the potential to be effective against *P. americana*. Bioassays revealed that CTD produces toxicity against *P. americana*. The median lethal concentration (LC_50_) value of CTD was 50.92 μg/mL, while NCTD displayed nearly no toxicity against *P. americana*. The inhibition assays of serine/threonine protein phosphatases (PSPs) in *P. americana* indicated that CTD and NCTD could inhibit PSPs. The value of the half maximal inhibitory concentration (IC_50_) of CTD was 7.21 ± 0.94 μM, whereas that of NCTD was higher, at 31.65 ± 3.87 μM. Furthermore, the inhibition effect of CTD on the serine/threonine protein phosphatase type 5 of *P. americana* (PaPP5) was superior to that of NCTD. Specifically, the IC_50_ of CTD reached 0.39 ± 0.04 μM, while the IC_50_ of NCTD was 1.87 ± 0.23 μM. This study paves the way for insect-derived agents (CTD) to be applied toward controlling *P. americana* and contributes to the development of novel insecticides based on PP5 as a target.

## 1. Introduction

The American cockroach, *Periplaneta americana* (L.), is a worldwide sanitary pest [1]. *P. americana* is of serious concern due to its ability to carry viruses, bacteria, and parasites [2]. It potentially contaminates food, thereby spreading disease and damaging public health [3]. This pest is difficult to control, since *P. americana* has both a strong reproduction capacity and survivability [4,5]. Currently, the control of cockroaches principally relies on chemical insecticides [6,7,8]. The nine classes of insecticides recommended by the WHO against cockroaches include carbamates, hydrazines, inorganics, insect growth regulators, neonicotinoids, organophosphates, pyrethroids, arylpyrazole, and sulfonamide [9,10]. However, previous reports have documented that cockroaches have developed resistance to some of these above-mentioned insecticides. For example, *P. americana* shows low levels of resistance against organophosphates and pyrethroids [11,12]. Therefore, the development of new agents is urgent in order to solve the insecticide resistance problem in controlling this cockroach.

Biopesticides have an edge over traditional chemical pesticides due to biopesticides being environmentally friendly and not prone to inducing resistance [13,14]. To date, many studies have reported botanical insecticides, such as essential oils, to have outstanding insecticidal activities. As reported, the *Curcuma longa* essential oil has been found to have an excellent insecticidal activity against *Lucilia cuprina* larvae, and a *Carlina acaulis* root essential oil has significant insecticidal activity against the housefly [15,16]. Meanwhile, research conducted to date shows that essential oils have effective contact, fumigation, and repellency activity against cockroaches [17,18]. In addition, the insecticidal activity of microbial agents and their metabolites on *P. americana* has also been extensively studied. Previous studies have shown that the application of mycoinsecticides for controlling *P. americana* has increased. *Metarhizium anisopliae*, *Metarhizium robertsii*, and *Beauveria bassiana* have been demonstrated to kill nymphs of *P. americana* [19,20]. However, little information is available on insect-derived pesticides for controlling cockroaches.

Cantharidin (CTD) comes from blister beetles, where it is a defensive toxin and protects blister beetles from attack [21,22]. More recently, its application in plant protection has attracted increasing attention. In the last century, there was a report indicating that CTD has the capacity to deter insects [23]. More recent reports indicate that CTD has contact, stomach, or repellent insecticidal toxicity against many pests, including *Plutella xylostella* (L.)*, Helicoverpa armigera* (Hübner), *Mythimna separate* (Walker), *Cydia pomonella* (L.), and *Musca domestica* (L.) [24,25,26,27,28,29,30]. Meanwhile, CTD also shows antifungal activity [31]. Previous research has demonstrated that CTD is an inhibitor of serine/threonine protein phosphatases (PSPs), including PP1, PP2A, PP4, PP5, and PP6, and the inhibition rate can reach nanomolar (nM) levels [32,33,34,35]. In addition, researchers have demonstrated that CTD is capable of inhibiting PP5 of *Plutella xylostella* (L.) [36]. The results produced by computer simulation support PP5 being the target of CTD in *P. xylostella* (L.), inferring that the insecticidal mechanism of CTD results from the inhibition of target protein PP5 [37]. However, the natural CTD resource is limited, and artificial synthetic versions are difficult to produce [38,39]. Therefore, the development of CTD analogues is of importance for its expanded application. Norcantharidin (NCTD) is a representative derivative of CTD and has a similar structure and bioactivity, as well as the same target protein as CTD (PSPs) [40]. In agriculture, NCTD has been found to have both insecticidal activity and antifungal activity [41].

In this study, the ingestion toxicity of CTD and its derivative NCTD against *P. americana* was determined through bioassays. In addition, we cloned the PP5 gene and expressed the catalytic domain of PP5 (PP5c) from *P. americana*. The inhibition capacity of CTD and its analogues on the PSPs and PP5c of *P. americana* were tested. The results indicate that CTD had a better toxicity on *P. americana* and the inhibition effect on the target protein PP5c was significant. These findings support the potential of CTD as a biorational pesticide to be applied to control *P. americana*, and may illuminate the application of insect-derived agents in controlling *P. americana*.

## 2. Materials and Methods

### 2.1. Periplaneta americana Strain

A susceptible *P. americana* strain without prior exposure to any insecticides or CTD was maintained in a glass chamber (80 × 32 × 30 cm) with sawdust in the bottom to maintain moisture. The glass chamber was smeared with Vaseline to prevent the *P. americana* from escaping. The rearing conditions were kept at 27 ± 2 °C, with a 65 ± 5% relative humidity and a photoperiod of 12:12 h (L:D). Breadcrumbs, milk powder, fresh apples, and water were supplied for *P. americana*. Healthy nymphs (emerging for 1–2 weeks) were used for the bioassays.

### 2.2. Chemicals

CTD was extracted and purified by a previously described method [42]. NCTD was purchased from Alfa Aesar (Haverhill, MA, purity > 98%) (Table 1).

### 2.3. Bioassays

All the agents were dissolved in acetone. The volumes of 2.5 mL of CTD (500 µg/mL) and NCTD (500 µg/mL) dilutions were each dropped in 2 g of breadcrumbs and fully mixed together. These bread crumb mixtures were then transferred into 50 mL conical flasks. Control groups were prepared with the same volumes of acetone. These treated conical flasks were kept in a fume hood to evaporate the acetone. Then nymphs were introduced to each conical flask after the evaporation of acetone. Absorbent gauze was used to seal the conical flask mouth. These conical flasks containing nymphs were then transferred to the above-mentioned conditions (27 ± 2 °C, 65 ± 5% relative humidity with a photoperiod of 12:12 h (L:D)). Each treatment was conducted in triplicate. The nymphs were judged to be dead if they lacked any movement after using a fine brush to poke their feet. The mortality was corrected by Abbott’s correction to ensure that the control morality remained lower than 15% [43]. The agents which could be effective against *P. americana* were serially diluted to perform the bioassay to determine the LC_50._ The values of LC_50_ in this bioassay were calculated as Probits using SPSS 20.0 (IBM, Armonk, NY, USA)

### 2.4. PSPs Activity and Inhibition Assay

Enzyme preparation: The crude PSPs enzyme was prepared according to the Serine/Threonine Phosphatase Assay System protocol (Promega, Cat. # V2460), with a slight modification. Ten nymphs were fully ground on ice in Tris-Hcl Buffer (pH = 7.4, containing 0.1% β-mercaptoethanol, 1 mM of EDTA, 1mM of PMSF, and 0.05% Triton-100). The homogenate was transferred into a 1.5 mL centrifuge tube and was centrifuged at 4 °C, 14,000 *g*, for 30 min. Then, 250 μL of supernatant was added to the Sephadex G-25 spin column and centrifuged at 4 °C, 600 *g*, for 5 min. The PSPs crude enzyme would then be in the bottom of the spin column.

PSPs activity assay: The phosphate standard curve was made following the protocol of the Serine/Threonine Phosphatase Assay System. In brief, the 1 mM phosphate standard solution was diluted with the supplied phosphate-free water into 50 μM. Different volumes (0, 2, 4, 10, 20, 40 μL) of diluted solution (50 μM) were separately added to a 96-well flat plate. The wells which were not 40 μL were supplied with phosphate-free water to achieve 40 μL. Then, 10 μL of buffer (containing 250 mM of imidazole, 1 mM of EDTA, 0.1% β-mercaptoethanol, 0.5 mg/mL of BSA, pH 7.2) was mixed with the 40 μL of diluted solution. The reaction mixture was incubated at 30 °C for 10 min. The reaction was stopped after the reaction mixture incubated with 50 μL of molybdate dye/additive mixture at 30 °C for 30 min. The absorbance of the samples was detected using an infinite M200 Microplate Reader (Tecan, Männedorf, Switzerland) at 600 nm. The obtained absorbance data were exported into Excel 2018 to establish the standard curve. The PSPs activity was measured using the Serine/Threonine Phosphatase Assay System kit (Promega, Madison, WI, USA). Then, 30 μL of phosphate-free water, 10 μL of assay buffer (containing 250 mM imidazole, 1 mM EDTA, 0.1% β-mercaptoethanol, 0.5 mg/mL BSA, pH 7.2), 5 μL of 1 mM phosphopeptide (RRA(pT)VA), and 5 μL of PSPs enzyme were added into the 96-well plate. The reaction mixtures were then fully mixed and incubated at 30 °C for 30 min. The reaction was terminated under the same conditions as described above. The absorbance at 600 nm (OD600) for each well was determined. Each assay was replicated three times. The amount of phosphate was calculated based on the phosphate standard curve.

Inhibition assay. The components of this measurement included 10 μg of PSPs protein and gradient concentrations of CTD or NCTD (100 nM–100 μM) dissolved in dimethyl sulfoxide (DMSO). This mixture was incubated for 10 min. Then, 25 μL of phosphate-free water, 10 μL of assay buffer, and 5 μL of 1 mM phosphopeptide (RRA (pT) VA) were added to the mixture. The reaction mixture was then incubated at 30 °C for 10 min. The reaction was stopped as stated before. The OD 600 of the samples was recorded by the above-mentioned Microplate Reader. The control groups used DMSO to replace CTD or NCTD. Each assay was replicated three times.

The relative inhibition was calculated using the Formula (1):

(1) $$\mathrm{Relative}\;\mathrm{inhibition}\;\left(\%\right)=\frac{\mathrm{OD}600c-\mathrm{OD}600t}{\mathrm{OD}600c}\times 100$$

OD600c: the OD600 of the control group; OD600t: the variation OD600 of the treatment group. Data were plotted into the prism using the GraphPad prism 6.0 software (GraphPad Software, Inc., La Jolla, CA, USA) to determine the value of IC_50_.

### 2.5. Molecular Cloning of P. americana pp5 (PaPP5)

Total RNA was extracted by RNAiso Plus (TaKaRa, Dalian, China) utilizing the manufacturer’s protocol. The concentration and the purity of extracted RNA was determined by NanoDrop-1000 (ThermoFisher, Waltham, MA, USA). First-strand cDNA was synthetized employing 1 μg of RNA using the RevertAidTM First-Strand cDNA Synthesis Kit (ThermoFisher,) following the instructions of the manufacturer, and stored at −20 °C. The known PP5 sequences of other insect species (*Diuraphis noxia*, GenBank: XP_015379688.1; *Acyrthosiphon pisum*, GenBank: XP_008181438.1; *Anoplophora glabripennis*, XP_018576896.1; *Pediculus humanus corporis*, XP_002425763.1; *Epicauta chinensis*, AHF45878.1, *Halyomorpha halys* XP_014290865.1, *Tribolium castaneum* XP_971407.1, *Riptortus pedestris* BAN20786.1; *Cephus cinctus*, XP_015610020.1; *Diachasma alloeum*, XP_015119779.1; *Orussus abietinus*, XP_012286908.1; *Neodiprion lecontei*, XP_015517121.1; *Monomorium pharaonic*, XP_012542571.1; *Apis mellifera*, XP_006567817.1; *Pseudomyrmex gracilis*, XP_020284073.1; *Apis dorsata*, XP_006613765.1; and *Linepithema humile*, XP_012231410.1) in NCBI GenBank were downloaded to design degenerate primers and amplify the conserved domain of PaPP5 (Table 2). The PCR process was operated on a S1000^TM^ thermal cycler (BioRad, Hercules, CA, USA). The amplified condition was followed by initial denaturation at 95 °C for 30 s, 30 cycles of denaturation at 95 °C for 30 s, annealing at 55 °C for 30 s and extension at 72 °C for 1 min, and with a final extension at 72 °C for 1 min. Any amplification fragment was identified by 1% agarose gel. The amplification product was extracted and purified using a Biospin Gel Extraction Kit (Biospin, Beijing, China). The purified PCR product was cloned into pMD-19T vector (TaKaRa, Dalian, China) and sequenced for confirmation. The protocol of the SMARTTM RACE cDNA Amplification Kit (TaKaRa, Dalian, China) was followed to synthesize 3′ and 5′RACE templates and conduct the amplification reaction. By applying the specific primers to amplify the hypothetical full-length *PaPP5*, we then cloned the fragment into pMD-19T vector (TaKaRa) and sequenced it for confirmation (Table 2). The ORF finder (https://www.ncbi.nlm.nih.gov/orffinder/) was then applied to determine the open reading frame (ORF).

### 2.6. Sequence Analysis of PaPP5

The multiple alignment of amino acids was acquired by DNAMAN 7.0 software (Lynnon Biosoft, San Ramon, CA, USA). The deduced molecular weight (Mw) and isoelectric point (pI) of PaPP5 were predicted using ExPASy web tools (http://www.expasy.org/). The active site and the metal binding site of PaPP5 were determined using InterPro (http://www.ebi.ac.uk/interpro/).

### 2.7. Recombinant PaPP5c Expression and Purification

The catalytic domain of PP5 (167-489 amino acids, PP5c) was ligated to the pMD-19T vector. The *PaPP5c*-pMD-19T plasmid was digested at the Nde1 and Xho1 site and then inserted into pET-30a (+) (TaKaRa) vector (digested at the same restriction sites) with T4 ligase (ThermoFisher). The recombinant plasmid was transformed into *E. coli* BL21 (DE3) cells. The positive transformant was cultured in LB liquid medium including 50 mg/mL of kanamycin at 37 °C with shaking at 200 rpm until the OD600 reached 0.5. We then added 0.1 mM of IPTG and 1mM of MnCl_2_ to induce protein expression at 22 °C, with shaking at 200 rpm for 24 h. Recombinant cells were centrifuged at 4 °C, 8000 *g*, for collecting the cell pellet. The harvested cell pellet was resuspended in PBS (10 mM), lysed with lysozyme (1 mg/mL), and then disrupted with sonication on ice for 10 min. After sonication, the supernatant was subjected to protein purification. The supernatant was purified with a Ni-NTA column (Smart-Lifesciences, Changzhou, China) using gradient concentrations of imidazole buffer (50–250 mM) to dissolve the protein. The purified protein was detected by 15% SDS-PAGE. Dialysate (4 mM of MnCl_2_, 10 mM of Tris-HCl, pH 8.0) was employed to dialyze the protein overnight at 4 °C. The concentration of purified protein was determined by the BCA method using the Easy II Protein Quantitative Kit (TransGene, Beijing, China).

### 2.8. Enzyme Activity Assay

The recombinant PaPP5c protein activity was measured in terms of DiFMUP as substrate (6,8-difluoro-4-methylumbelliferyl phosphate) with a slight modification [44]. The reaction mixture contained 1 μg of recombinant PP5, 10 μL of different concentrations of DiFMUP (1, 3, 6, 12, 25, 50, and 100 μM), and 90 μL of buffer (containing 30 mM of HEPES, 0.1 mg/mL of BSA, 0.1 mM of MnCl_2_, 1 mM of sodium ascorbate, 1 mM of DTT, and 0.01% triton X-100). The components of this reaction mixture were added into a black 96-well plate (ThermoFisher, USA) in sequence. The reaction mixture was incubated for 30 min at 30 °C. Then, the fluorescence values were recorded using an Infinites 200 PRO multimode micro-plate reader (Tecan, Männedorf, Switzerland) (excitation at 360 nm, emission at 450 nm). The inactivated protein supplanted the enzyme as a control group. Data were plotted using GraphPad Prism 6.0 (GraphPad Software) to determine the enzyme kinetics with the Michaelis–Menten option. Each assay was replicated three times.

### 2.9. In Vitro Inhibition Assays on PP5c

The test agents were dissolved in a 10 mg/mL stock solution with DMSO. The stock solution was diluted in gradient concentrations with water in later inhibition assays. Initially, 1 μg of recombinant PP5c, 1 μL of CTD or NCTD, and 100 μL of reaction buffer (30 mM of HEPES, 0.1 mg/mL of BSA, 0.1 mM of MnCl_2_, 1 mM of sodium ascorbate, 1mM of DTT, 0.01% triton X-100) were incubated for 10 min at 30 °C. Afterwards, 10 μL of DiFMUP (100 μM) was added into the reaction mixture for incubation for 30 min at 30 °C. The reaction was stopped by adding 100 μL of 300 mM phosphate (pH 10). The inactivated protein replaced the enzyme as a negative control group. Meanwhile, the positive control group used DMSO in place of the recombinant protein. Each assay was replicated five times. The fluorescence values for the inhibition assay are the same as with the enzyme activity assay.

Relative inhibition was calculated using the Formula (2):

(2) $$\mathrm{Relative}\;\mathrm{inhibition}\;\left(\%\right)=\frac{\mathrm{control}\;\left(\mathrm{Fv}\right)-\mathrm{treatment}\left(\mathrm{Fv}\right)}{\mathrm{control}\;\left(\mathrm{Fv}\right)}\times 100.$$

Control (Fv): the fluorescence value of the control group; treatment (Fv): the fluorescence value of the treatment group. Data were plotted into prism using the GraphPad prism 6.0 software (GraphPad) to determine the value of IC_50_.

## 3. Results

### 3.1. Toxicity of CTD and Analogues on P. americana

Under the primary screening concentration of 500 µg/mL, CTD resulted in a 100% mortality rate. The mortality rate from NCTD was far below that for CTD. The mortality rate caused by NCTD was 13.33%. (Table 3). The results of toxicity regression equations show that CTD caused the most significant toxicity, and the LC_50_ was 50.92 µg/mL for LC_50_ (Table 4).

### 3.2. Inhibition Effects of CTD and NCTD on PSP of P. americana

**Figure 1:**
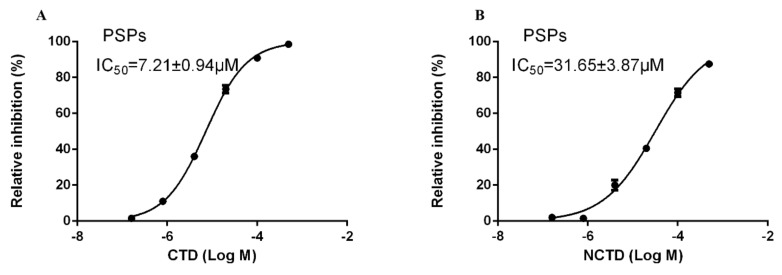
Inhibition curves of Cantharidinand Norcantharidin on the serine/threonine protein phosphatase (PSPs) of *P. americana*. Data are shown as mean ± SD. (**A**) Cantharidin, (**B**) Norcantharidin.

An in vitro inhibition assay of CTD and NCTD on the crude PSP of *P. americana* displayed CTD, and NCTD had inhibitory effects on PSP, whereas the differences in the values of IC_50_ were obvious. The inhibition effect of CTD was superior to that of NCTD. The IC_50_ of CTD was 7.21 ± 0.94 μM, whereas the value of NCTD (31.65 ± 3.87 μM) was over four times that of CTD (Figure 1).

### 3.3. Characterization of PaPP5 Sequence

**Figure 2:**
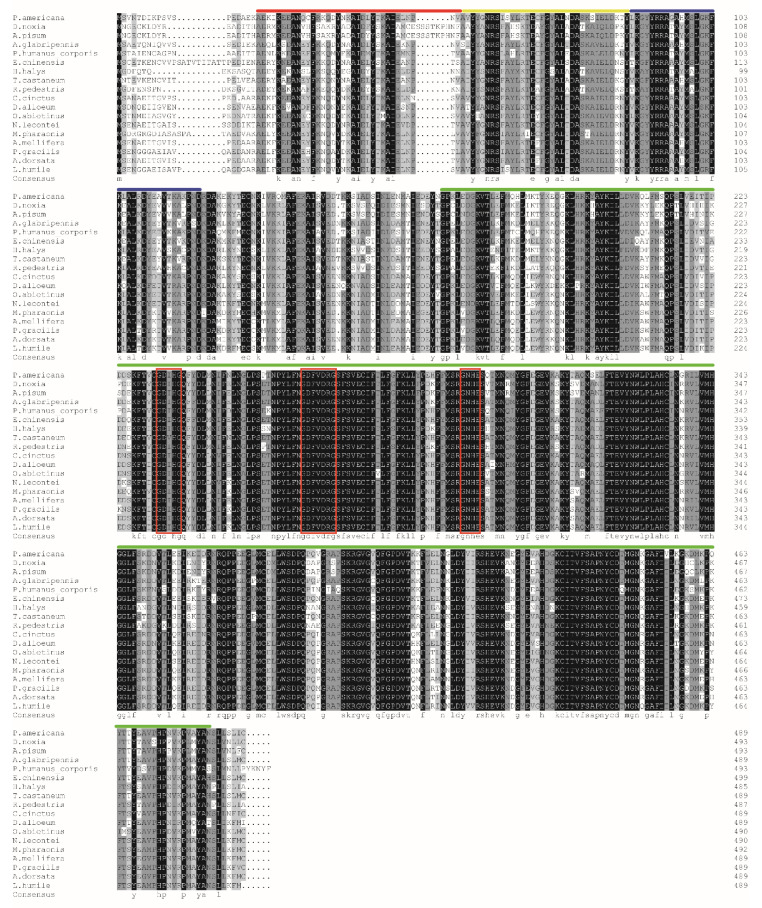
Multiple alignments of the deduced amino acid sequences of PP5 from *P. americana* and with other insect species ((*D. noxia*, GenBank: XP_015379688.1; *A. pisum*, GenBank: XP_008181438.1; *A. glabripennis*, XP_018576896.1; *P. humanus corporis*, XP_002425763.1; *E. chinensis*, AHF45878.1, *H. halys* XP_014290865.1, *T. castaneum* XP_971407.1, *R. pedestris* BAN20786.1; *C. cinctus*, XP_015610020.1; *D. alloeum*, XP_015119779.1; *O. abietinus*, XP_012286908.1; *N. lecontei*, XP_015517121.1; *M. pharaonic*, XP_012542571.1; *A. mellifera*, XP_006567817.1; *P. gracilis*, XP_020284073.1; *A. dorsata*, XP_006613765.1; *L. humile*, XP_012231410.1). The TPR domains are shown in red, yellow, and blue lines. The predicted catalytic domain of PaPP5 (PP5c) is indicated by a green line. The three conserved motifs (-GDXHG-, -GDXVDRG-, and -GNHE-) are presented in a red horizontal rectangle.

We cloned the full-length cDNA of PaPP5 and uploaded the amino acids sequence of ORF to GenBank. The obtained accession number was AZT88974.1. Multiple alignment shows that PaPP5 shares an 80.81% identity with the compared PP5 of different insect species. The deduced full-length ORF sequence of PaPP5 encoded 489 amino acids. The predicted theoretical molecular weight (Mw) and isoelectric point (pI) were 55.82 KDa and 6.18, respectively. Additionally, the predication based on the obtained PaPP5 sequence displays that the predicted catalytic domain of PaPP5 (PP5c) consists of the 167–482 residues and the TPR (tetratricopeptide repeat) are from 19–52, 53–86, and 87–120 amino acids (Figure 2). The deduced molecular weight of PaPP5c was 36.09 KDa. The three conserved motifs (-GDXHG-, -GDXVDRG- and -GNHE-) of the PSPs catalytic domain in PaPP5 were GDIHG, GDFVDRG and GNHE.

### 3.4. The Recombinant PaPP5c

**Figure 3:**
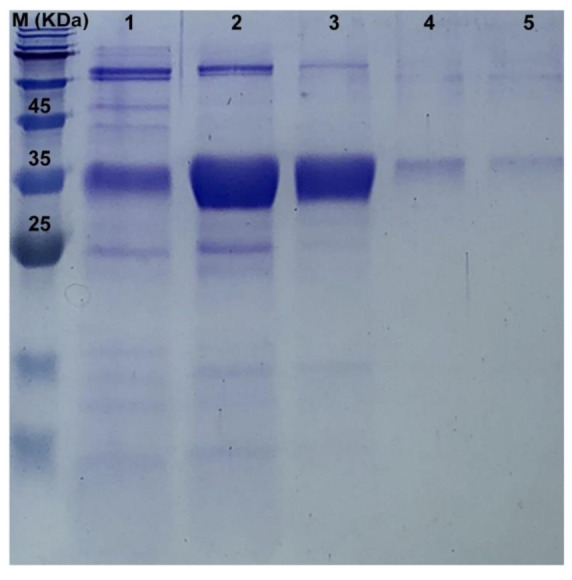
Analysis of the recombinant PaPP5c by 15% SDS-PAGE. M: protein marker; Lane 1: 50 mM imidazole elution; Lane 2: 100 mM imidazole elution; Lane 3: 150 mM imidazole elution; Lane 4: 200 mM imidazole elution; Lane 5: 250 mM imidazole elution.

**Figure 4:**
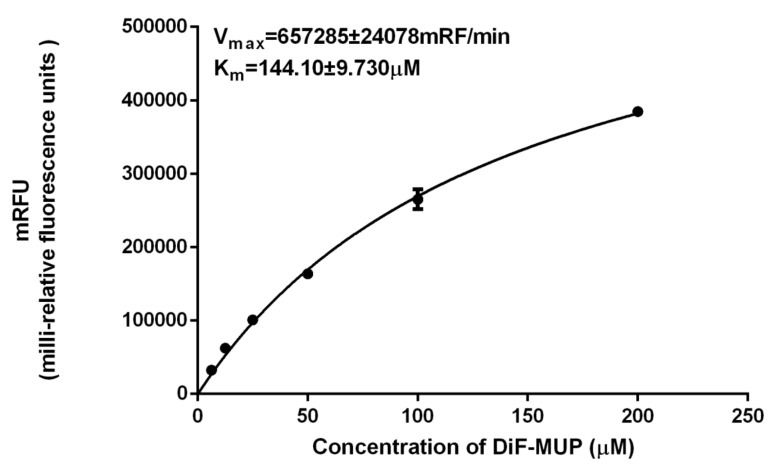
Kinetics of DiFMUP (6,8-difluoro-4-methylumbelliferyl phosphate) dephosphorylation by the catalytic domain of PP5 (PP5c). Data are shown as mean ± SD.

The N-terminal region of PP5 has an autoinhibitory function and thus inhibits the catalytic activity [45]. Therefore, we expressed PP5c in vitro. SDS-PAGE showed that we obtained the fusion recombinant PP5c, which was consistent with the predicated molecular weight (36.89 kDa) calculated from the deduced amino acid sequence of PaPP5c with His-tag in pET-30a (Figure 3). The concentration of recombinant PaPP5c was 0.5 μg/μL. According to the Michaelis−Menten plot, the k_m_ was 144.10 ± 9.730 μM and the V_max_ of the recombinant PP5c was 657,285 ± 24,078 mRF/min, respectively (Figure 4)

### 3.5. Inhibition Effects of CTD and NCTD on PaPP5c

**Figure 5:**
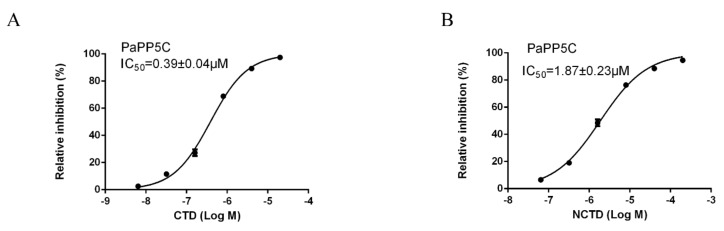
Inhibition curves of Cantharidin and Norcantharidin on the catalytic domain of PP5 (PP5c). Data are shown as mean ± SD. (**A**) Cantharidin, (**B**) Norcantharidin.

We determined the inhibition effects of CTD and NCTD on PaPP5c. The results show that CTD has a more significant inhibition effect on PaPP5c compared to NCTD. The IC_50_ of CTD on PaPP5c is 0.39 ± 0.04 μM. NCTD was inferior to CTD, resulting in an IC_50_ of 1.87 ± 0.23 μM (Figure 5).

## 4. Discussion

Currently, insecticide resistance remains one of the barriers for agricultural production and public health pest management. Therefore, the development of novel insecticides is needed. CTD has been previously reported to have insecticidal activity in a broad range of pests [29]. However, CTD has not been widely applied in the field due to the high cost of acquisition. In this study, we determined the ingestion toxicity of CTD and its derivative NCTD on *P. americana*. The results illustrate that CTD displays a significant toxicity against *P. americana*. This agrees with a previous report documenting how CTD had significant insecticidal activity against many pests [29]. In addition, this study demonstrates that it has a similar toxicity against *P. americana.* Meanwhile, this result also suggests that CTD may have the potential to be effective against a broader range of pests. Additionally, although previous studies have reported that NCTD shows an excellent toxicity on *P. xylostella*, it did not show any significant toxicity on *P. americana*. We infer that this difference probably results from either different feeding or the presence of different penetration and metabolic characteristics between different species. Although both CTD and NCTD showed strong inhibitory effects on PSPs, their toxicity against *P. American* displayed huge differences. This may due to the inhibition on PSPs is not the sole mechanism for toxicity. Additionally, the huge disparity in toxicity between NCTD and CTD may result from differences in the structure. The changes in structure may alter the metabolic pathway of *P. americana* towards NCTD. Furthermore, some gut microorganisms may be involved in the detoxification of NCTD.

Previous research has demonstrated that CTD can inhibit PSPs [34]. To verify the effects of the CTD and NCTD on PSPs in *P. americana*, we conducted inhibition assays on PSPs in vitro. In vitro inhibition assays indicate that the activity of PSPs is significantly inhibited by CTD and NCTD. As reported, CTD can effectively inhibit the activity of PSPs in plants such as *Arabidopsis thaliana* (IC_50_ = 0.63 μM) [33]. It also has been reported that CTD can inhibit the activity of PSP_S_ in *P. xylostella* (IC_50_ = 5.39 μM) [36]. In this study, the value of IC_50_ of CTD on PSPs was 7.21 μM, which is comparable to the above-mentioned values of IC_50_. This indicates that CTD has a strong inhibition effect on PSPs in *P. americana*. Information on the analogue of CTD on PSPs of insects is little known. In the present study, NCTD showed inhibition effects on PSPs, indicating that their potential inhibition capacity can be comparable to that of CTD. Our results show that CTD and NCTD have the capacity to inhibit the activity of PSPs in *P. americana*. These results indicate that CTD and NCTD have strong inhibition effects on the PSP family of proteins of *P. americana*. However, it is not clear which specific PSPs in *P. americana* have their activity inhibited by CTD and NCTD.

The PSPs protein family encompasses a large number of members, including PP1, PP2A, PP2B, PP4, PP5, PP6, and PP7 [46]. In our previous study, we evaluated the binding mode of the interaction between the PxPP5 of *P. xylostella* and CTD, indicating that CTD had a strong binding affinity towards PP5 [36]. Therefore, we propose that the toxicity of CTD and its analogues against *P. americana* is also due to their inhibition effects on PP5. It has been reported that PP5 is comprised of an N-terminal tetratricopeptide repeat (TPR) and a C-terminal region [47]. The catalytic domain of PP5 (PP5c) is highly conserved and shares a consensus sequence (-GDXHG-, -GDXVDRG-, and -GNHE-) with other PSP members [34]. Here, we cloned the PP5 of *P. americana*. The sequence analysis shows that PaPP5 shares a highly conserved TPR and catalytic domain (PP5c), which is consistent with earlier reports of the characteristics of the PP5 structure. Furthermore, we obtained the fusion recombinant PaPP5c via prokaryotic expression, suggesting that the expression conditions for PaPP5c are suitable in this study. Additionally, previous reports on the kinetics of Human PP5c are as follows: Km: 141 ± 10.7 μM; Vmax: 216,9730 ± 49,180 mRFU/min [43]. The results of the kinetics of recombinant PaPP5c demonstrate that the recombinant PaPP5c were comparable to the previously reported result, and thus the obtained purified recombinant PaPP5c could perform the following tests. According to a previous report, the IC_50_ of CTD on human PP5c can reach 0.2 μM [48]. In this study, the values of the IC_50_ of CTD and NCTD on PaPP5c also reached 0.39 and 1.87 μM, respectively, showing an inhibitory effect on PaPP5c. Additionally, the results of the inhibition assay on PP5c further support the bioassay results. CTD shows the most significant inhibition rates on PP5c, and this result corresponds to the bioassay showing that CTD induced the most ingestion toxicity on *P. americana*. While the inhibition rate of NCTD on PaPP5c is obviously inferior to that of CTD, its results in the bioassay are far inferior to those of CTD. As reported, PP5 is involved in cellular survival and death. Meanwhile, PP5 is vital for regulating signaling, which regulates a series of physical functions such as metabolism [49]. Because of the strong inhibition effects of CTD on PaPP5c, the metabolism may be disrupted. Therefore, we infer that the toxicity of CTD on *P. americana* is likely due to its inhibition effect on PP5c. Although NCTD did not show significant toxicity against *P. americana*, it suggested that the CTD derivatives which have a structure like that of NCTD may not compare to CTD. However, considering that CTD showed an excellent toxicity against *P. americana*, it can be inferred that other derivatives of CTD which do not have an NCTD-like structure may have a similar bioactivity to that of CTD. In this way, the development of CTD derivatives is of significance.

## 5. Conclusions

Overall, the above findings demonstrate that CTD has insecticidal activity against *P. americana*. The inhibition effect of CTD on PaPP5c may be responsible for the toxicity of CTD towards *P. americana*. This study promotes CTD as a potential agent to control *P. americana*, and this may contribute to the development of CTD derivatives for deterring pests. In addition, considering how PP5 is affected by CTD and its analogue, we speculate that PP5 may be a novel target for future insecticide development.

## Figures and Tables

**Table 1:** Chemical structural formula and identifier of cantharidin and analogues.

Chemical Structural Formula	Identifier	Chemical Structural Formula	Identifier
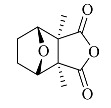	Cantharidin	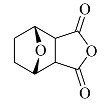	Norcantharidin

**Table 2:** Primers used in this study.

Gene	Primers	Sequence (5′–3′)	Usage
*PaPP5*	PaPP5D-F	CACATGTCCCTGGGCAAGTWYAARYTNGC	Degenerate primers
	PaPP5D-R	GGNAARTGYATCACCGTGTTCTCCGCC	Degenerate primers
*PaPP5*	5PaPP5-1	TGGAACTTCTTGTGGGCGA	5′ RACE
	5PaPP5-2	GAATAATATCCACGGTCAACA	5′ RACE
	3PaPP5-1	GAAGGTGAAGTAAAGGCGAAG	3′ RACE
	3PaPP5-2	GAAATAGGCAACCTCCAGAAG	3′ RACE
*PaPP5c*	PaPP5c-F	CCCATATGCACCACCACCACCACCACTACAGCGGACCCAAGCTTG Nde1	Amplification of PaPP5c
*PaPP5c*	PaPP5c-R	CCCTCGAGTCACATCATTCCTAGCTGC Xho1	Amplification of PaPP5c

**Table 3:** Toxicity of cantharidin and its analogue to *P. americana* at 500 µg/mL.

Identifier	Mortality Rate (%)
Cantharidin	100
Norcantharidin	13.33

**Table 4:** Toxicity determination of effective compounds on *P. americana* (48 h).

Identifier	Toxicity Regression Equations	LC_50_ (µg/mL)	χ^2^	95% Confidence Interval
Cantharidin	Y= −5.611 + 3.326X	50.920	1.998	19.204–82.211
